# Motor resonance facilitates movement execution: an ERP and kinematic study

**DOI:** 10.3389/fnhum.2013.00646

**Published:** 2013-10-15

**Authors:** Mathilde Ménoret, Aurore Curie, Vincent des Portes, Tatjana A. Nazir, Yves Paulignan

**Affiliations:** ^1^Laboratoire sur le Langage, le Cerveau et la Cognition L2C2, Centre National de la Recherche Scientifique/UCBL, UMR 5304, Institut des Sciences CognitivesLyon, France; ^2^Service de Neuropédiatrie, Hospices Civils de Lyon, HFMELyon, France

**Keywords:** motor resonance, mirror neurons, kinematics, EEG, motor system

## Abstract

Action observation, simulation and execution share neural mechanisms that allow for a common motor representation. It is known that when these overlapping mechanisms are simultaneously activated by action observation and execution, motor performance is influenced by observation and vice versa. To understand the neural dynamics underlying this influence and to measure how variations in brain activity impact the precise kinematics of motor behavior, we coupled kinematics and electrophysiological recordings of participants while they performed and observed congruent or non-congruent actions or during action execution alone. We found that movement velocities and the trajectory deviations of the executed actions increased during the observation of congruent actions compared to the observation of non-congruent actions or action execution alone. This facilitation was also discernible in the motor-related potentials of the participants; the motor-related potentials were transiently more negative in the congruent condition around the onset of the executed movement, which occurred 300 ms after the onset of the observed movement. This facilitation seemed to depend not only on spatial congruency but also on the optimal temporal relationship of the observation and execution events.

## Introduction

Action observation, simulation and execution share neural mechanisms that allow for common motor representation (Prinz, 1997; Jeannerod, 2001). Mirror neurons, initially discovered in monkeys, may represent a correlate of this “action observation-execution” matching system (Gallese and Goldman, 1998). Indeed, these mirror neurons, which have been identified in the macaque ventral premotor cortex and inferior parietal lobule, fire during the execution of an action but also during the observation of the same action (Di Pellegrino et al., 1992; Gallese et al., 1996; Rizzolatti et al., 2001; Umiltà et al., 2001). Such mirror activities have also been described in human premotor and parietal cortices using functional magnetic resonance imaging (fMRI) and electroencephalography (EEG) (Buccino et al., 2001; Pineda, 2008; Kilner et al., 2009), leading to the hypothesis that there is a human “mirror neuron system” (MNS). Recently, using extracellular neuronal recordings, Mukamel et al. (2010) identified single neurons with mirror properties in the human supplementary motor area (SMA), the hippocampus, the parahippocampal gyrus and the entorhinal cortex. These regions, which are not classically described as being a part of the mirror system, may provide evidence that the MNS is more dispersed than initially thought (Keysers and Gazzola, 2010). Taken together, these findings suggest that observing an action and performing the same action activate overlapping networks, which raises the question of what happens when these events occur simultaneously, i.e., in joint action situations. Joint actions involve at least two participants acting together, simultaneously or alternatively to achieve a common goal (Sebanz et al., 2006). Therefore, coordinated behaviors are essential for successful interaction, and we may consider the implication of the Action-Observation network in such coordination and the importance of the optimization of the temporal coordination between observed and executed actions (Knoblich et al., 2011).

It has been hypothesized that the co-activation of motor structures during execution and observation could result in a “motor resonance system” that may influence both the execution and the perception of the action (Blakemore and Frith, 2005). Several behavioral studies have tested this hypothesis (Brass et al., 2000, 2001a; Kilner et al., 2003; Casile and Giese, 2006; Schütz-Bosbach and Prinz, 2007; Stanley et al., 2007; Ramsey et al., 2010; Christensen et al., 2011). These studies tend to show that the simultaneous activation of the overlapping neural networks that process both movement observation and execution confers a measurable cost to motor control (Blakemore and Frith, 2005). For example, Kilner et al. (2003) asked participants to perform arm movements while observing another person making the same (congruent) or qualitatively different (non-congruent) arm movements (horizontal or vertical). Greater variability was noted when participants observed someone performing incongruent movements compared to performing congruent ones. However, it also seems that observing an action can facilitate action execution. Brass et al. (2000), for instance, asked participants to perform finger movements while observing congruent or non-congruent movements. Although these authors observed an interference effect [longer reaction times (RT)] when participants were observing non-congruent movements, they found facilitation when participants observed congruent finger actions (shorter RTs).

Some studies have also investigated the complementary impact of the execution of an action on movement perception and observed facilitation or interference effects (Casile and Giese, 2006; Cattaneo et al., 2011; Barchiesi et al., 2012). This modulation has been referred to as “perception resonance” by (Schütz-Bosbach and Prinz, 2007) or as a motor-to-visual after-effect by others (Cattaneo et al., 2011; Barchiesi et al., 2012). Motor preparation (Fagioli et al., 2007), movement execution (Miall et al., 2006; Cattaneo et al., 2011; Barchiesi et al., 2012) and motor learning without visual feedback (Casile and Giese, 2006) can thus, improve perceptual performance (e.g., movement discrimination). This effect may depend on the modulation of visuo-motor neurons in the premotor cortex, and this modulation may prime perception by selectively facilitating the recognition of related actions or actions that share features with the executed actions (Cattaneo et al., 2011). Taken together, all these findings emphasize the tight links between action and perception. However, little is currently known about the temporal dynamics of this cross-talk.

Catmur et al. (2011), Christensen et al. (2011) and Ménoret et al. (2013) have demonstrated that the motor resonance effect is sensitive to the temporal delay between movement execution and observation. For example in a TMS study, Catmur et al. (2011) showed that a delay of 250–300 ms between action observation and execution is crucial for the induction of a facilitation effect when stimulating the premotor cortex. Similarly, in one of our previous experiments, we could show that the temporal relationship of observation and execution is critical for the emergence of motor resonance (Ménoret et al., 2013). In this experiment, we tested whether the observation of a grasping action (directed toward a small or a large object) affects the execution of a congruent or non-congruent action. The observed action could occur shortly (200 ms) or well before (1 s) the onset of movement execution. We found that observing a congruent grasping action optimized the grasp component of the movement (i.e., the maximal grip aperture) only when the movements occurred within a 200 ms delay and not when they were separated by 1 s (Ménoret et al., 2013). Finally, Christensen et al. (2011) found that interference or facilitation effects can be manipulated by the temporal relationship of movement execution and observation. Participants were asked to recognize a point-light stimulus controlled by their own movements in a scrambled mask during the execution of waving movements. The results of these authors showed that the identification of the movement was facilitated when the movement to be identified was congruent and synchronized with the movement being executed. Conversely, identification was disturbed when the movement to be identified was non-congruent or not synchronized with the performed movement (Christensen et al., 2011). These studies, and especially those by Catmur et al. (2011) and Ménoret et al. (2013), thus, indicate that the optimal delay between observation and execution for maximizing facilitation should be approximately 200–300 ms.

Few studies have attempted to investigate the neural correlates of such contagion effects. Brass and colleagues proposed that the interfering effects may result from an inhibitory mechanism that prevents automatic motor responses (Brass et al., 2001b). In an fMRI study, these authors showed that during incongruent trials, prefrontal and parietal cortices were more activated than during congruent trials. Similarly, Stanley and Miall (2007) reported enhanced activities in non-congruent trials in the superior parietal lobules and the dorsal premotor cortex. Thus, these results indicate that these areas may be involved in such inhibitory mechanisms that prevent the execution of an imitative response (Brass et al., 2001b). However, no studies have investigated the time course of this motor resonance largely because of the low temporal resolution of fMRI. In an ERP experiment, Van Schie et al. (2004) analyzed the neural mechanisms of error processing during action observation. These authors found that the error-related negativity (ERN) as well as the lateralized readiness potential (LRP), which is a brain potential that is thought to reflect the preparation of motor activity, were modulated by the accuracy of the actor's observed response. Observing the correct response induces an LRP that is similar to the LRP observed during the actual movement; by contrast, when the actor made an error the observer's LRP was less important. However, no effects were observed on the observer's electromyogram, and this study did not investigate the effect of simultaneous observation and execution.

The aim of the present study was to determine how the simultaneous observation and execution of actions influences the processing of others' actions and the execution of one's own actions. Indeed, in joint action situations during simultaneous actions (e.g., lifting a table), your partner's actions may influence your own actions to achieve successful coordination. However, the temporal dynamics of this influence are still poorly known.

Using a fine-grained kinematic analysis coupled to high temporal resolution EEG, we sought to confirm the temporal profile of “motor facilitation” in the context of real observations. During the observation of an actor's action, we measured how observers' behaviors and cerebral activities were modulated by the congruency of the observed action. For this, we modified the initial experiment of Kilner et al. (2003) described above and used a 300 ms delay between execution and observation, identified as optimal delay by previous studies (Catmur et al., 2011; Ménoret et al., 2013). Recordings of movement kinematics and the EEG provided us with the opportunity to analyse the precise kinematics of the movements (velocity, duration, and trajectory) and to directly synchronize the electrophysiological events recorded from the observer with the outcome of the actor's motor output (i.e., the onset of visual information for the observer).

Thorough analyses of standard kinematics parameters enabled us to precisely and completely describe how movement performance was influenced by the observation of an actor's movement. We expected to observe a facilitation of movement execution as indicated by earlier and stronger velocity peaks, shorter movement durations and optimized trajectories.

The concurrent analysis of motor related potentials (MRP) that are time-locked to the onset of the actor's movement, further allowed us to describe the time course of the motor contagion and the exact duration of the optimal time-window for inducing facilitation.

## Methods

### Participants

Seventeen healthy participants [mean age: 23.7 (range: 19–35 years), 6 women and 11 men] took part in this experiment. They reported no history of neurological disease or psychological issues. All were right handed [mean scores: 0.76 Edinburgh test (Oldfield, 1971)] and had normal or corrected-to-normal vision. The study was approved by the Ethical Committee CPP Sud-Est II and all participants gave their written informed consent. An unknown partner was assigned to each participant.

### Procedure

**Figure 1:**
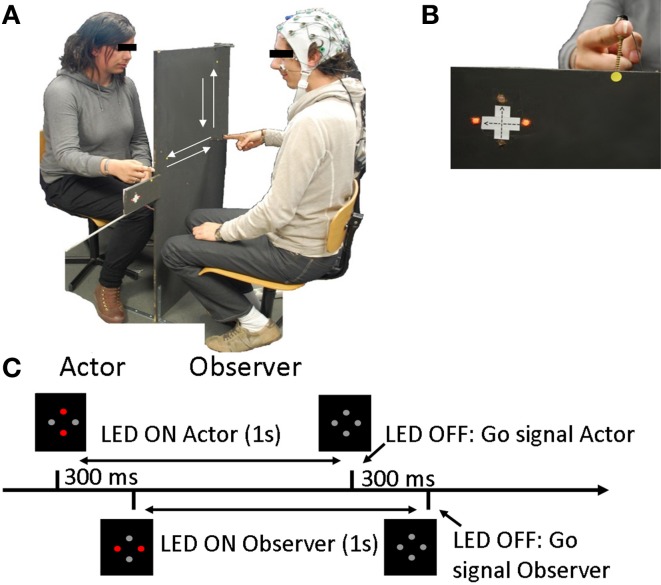
**Experimental design. (A)** Experimental setup including an Actor and an Observer. **(B)** Observer's view, including the point of fixation (yellow dot), the instructions (LED) and the actor's hand. **(C)** Trial setup: Instructions were displayed with the red LED and the LED switched off 1 second later, signifying the “Go” signal. The Actor's instructions and the “Go” signal were given 300 ms before the observer's.

The experiment always involved a pair of participants, one referred to as the “observer” and the other as the “actor.” Movement kinematics of both participants and the observer's EEG were recorded throughout the experiment. Pairs of participants were seated in front of each other. The actor was placed behind a black panel that prevented him from seeing the observer. In contrast, the observer could see the actor's hand. Thus, the observer could be influenced by the actor's movements whereas the actor could not. Figure 1A displays the protocol.

The pair of participants had to execute linear back-and-forth arm movements in either the horizontal (H) or vertical (V) direction. The movements consisted of one back-and-forth arm movement cycle approximately 40 cm long beginning from a fixed starting point on the panel. The experiment included 3 different conditions (including two sub-conditions):

– *Execution only (control condition)*: the observer performed either a horizontal (EH) or a vertical (EV) movement and the actor did not perform any movement.
– *Congruent condition*: the observer and the actor performed movements in the same direction, either Horizontal (HC) or Vertical (VC).
– *Non-Congruent condition*: the observer performed a Horizontal (HNC) or Vertical (VNC) movement while the actor performed the movement in the other direction.

Each sub-condition was presented 25 times in a pseudo-randomized order. In total, the experiment was composed of 150 trials lasting approximately 5 s.

Instructions were displayed with 4 red LED lights (Figure 1B) that were affixed to each side of the panel, visible only to one participant.

At the beginning and the end of each trial, both participants were asked to place their right index fingers on the starting position. Participants were instructed to begin their movements only when the lights (instruction) switched off (“Go” instruction). The actor's “Go” signal always occurred 300 ms before the observer's, and therefore, the actor always began his movement before the observer, regardless of the observer's RT. Each trial proceeded as presented Figure 1C: instructions were displayed in front of the participants for 1 s, 300 ms after the actor. The actor's instructions switched off 300 ms before the observer's, signaling the beginning of the trial.

To minimize the observer's eye movement artifacts, a fixation point was placed between the LED and the actor's hand. Thus, both lights and hands were in his/her visual field. The observer was asked to maintain fixation throughout the trial and thus, to observe the actor's movements with peripheral vision.

### Kinematics acquisition and analysis

An Optotrak 3020 (Northern Digital Inc., Waterloo, Ontario Canada) was used to record the spatial position of an active marker (infrared light-emitting diode) at a sampling rate of 250 Hz and a spatial resolution of 0.1 mm. The marker was placed on the participant's index finger. Raw data were pre-processed using a second-order Butterworth dual-pass filter (cut-off frequency, 10 Hz). Kinematic parameters were assessed for each individual movement using Optodisp software [Optodisp Copyright INSERM-CNRS-UCBL (Marc Thevenet, Yves Paulignan, Claude Prablanc) 2001].

For the two sub-phases of the movement (back and forth), we analyzed the amplitude (Vel_1_ and Vel_2_), and the latency (LatPeak_1_ and LatPeak_2_) of the index velocity peaks (mm/s) as well as movement durations (ms) (Duration_total_, Duration_1_, and Duration_2_). The coordinates of the extrema of the trajectory (on x, y, and z axis) and the RT were also analyzed. Movement onset and offset were determined to be the first and last, respectively, value of a sequence of at least eleven increasing or backward increasing, respectively, points on the basis of the wrist velocity profile. Wrist velocity peak was determined as the maximal value in the velocity profile. Kinematic parameters were determined for each individual trial and were then averaged for each participant and condition. All error trials were removed from the analysis.

Repeated-measures ANOVA (Direction × Condition) was performed on all of these parameters to compare the conditions for the actor and the observer separately.

For each individual trial, the latency of the onset of the participant's movement was determined to synchronize the observer's EEG with the onset of the actor's movement.

### EEG acquisition and analysis

EEG data were recorded using BrainAmp amplifiers (BrainVision recorder software, BrainProducts GmbH, Munich, Germany). The observer's EEG data were recorded with an EEG device using 32-channel EEG caps with active electrodes (ActiCap BrainProducts) placed according to the international 10–20 system. Reference and ground were situated at Fpz and AFz, respectively, and impedance was maintained below 20 kΩ. The signal was sampled at 500 Hz and a 50 Hz notch filter was used. Moreover, recordings of vertical and horizontal electro-oculograms (EOGs) were made from electrodes above and below the left eye to monitor eye movements and blinks.

EEG data were analyzed using BrainProducts Analyser 2 software (BrainProducts GmbH, Munich, Germany). EEG data were time-locked to the onset of the actor's movements by adding kinematic markers to the EEG signal. It was then re-referenced with a mean reference value including all but the EOG electrodes and was low pass-filtered at 30 Hz. EEG data were segmented from 1000 ms before the onset of the actor's movement to 1000 ms after the onset.

After segmentation, a baseline correction was applied from −200 to 0 ms before the onset of the actor's movement. Averages were calculated within the four sub-conditions (HC, HNC, VC, VNC). Grand averages for all participants were calculated separately for each condition.

### Motor related potentials/statistical analysis

ERPs signal were synchronized on the onset of the actor's movement. This synchronization that allow a good precision for movement analysis, does not allow us to characterize precisely a Contingent Negative Variation (CNV) (traditionally time-locked to the Go signals) (Libet, 1985; Leuthold et al., 2004). Therefore, this negative ongoing wave will be referred to as a MRP.

The MRP (Readiness potential, CNV) are commonly described in central sites (Libet, 1985; Leuthold et al., 2004). Therefore, the Cz electrode was defined as the region of interest for the MRP.

To determine a time window of interest, an exploratory analysis was first performed. A standard *t*-test was used to compare Congruent and Non-Congruent conditions for each 2 ms time interval from 0 to 1000 ms after the actor's movement onset. A 100 ms time window was selected and the average amplitudes of the ERPs were computed for each condition within this time-window. A repeated-measures ANOVA (Direction × Condition) was performed to compare the four sub-conditions.

## Results

### Behavioral results

A preliminary analysis was conducted to verify whether the actors' movements were comparable between the conditions. The results are reported in Supplementary Table 1. A repeated-measures ANOVA (Direction × Condition) did not reveal a significant effect of Condition (Congruent and Non-Congruent) on any of the parameters: velocity peaks of the index (Vel_1_ and Vel_2_), movement duration (Duration_1_, Duration_2_, Duration_total_), the coordinates of the extrema of the trajectory (on x, y and z axis) and RT (see Supplementary Table 1).

**Figure 2:**
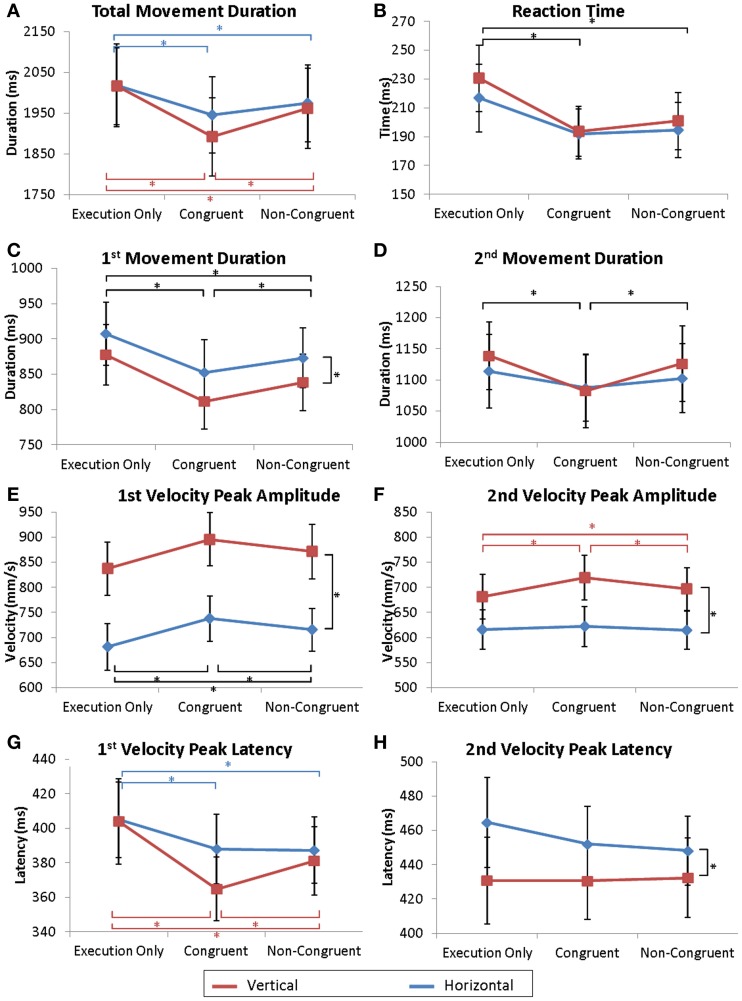
**Kinematic parameters of the movement**. Mean values of total movement duration **(A)**, reaction times **(B)**, 1st and 2nd parts of the movement duration **(C,D)**, 1st and 2nd velocity peak amplitudes **(E,F)** and latencies **(G,H)**. Error bars represent the standard error of the mean (s.e.m.). ^*^p = 0.05. Dark lines correspond to a main effect of condition whereas colored lines correspond to specific effects of horizontal (red) or vertical (blue) movements.

**Figure 3:**
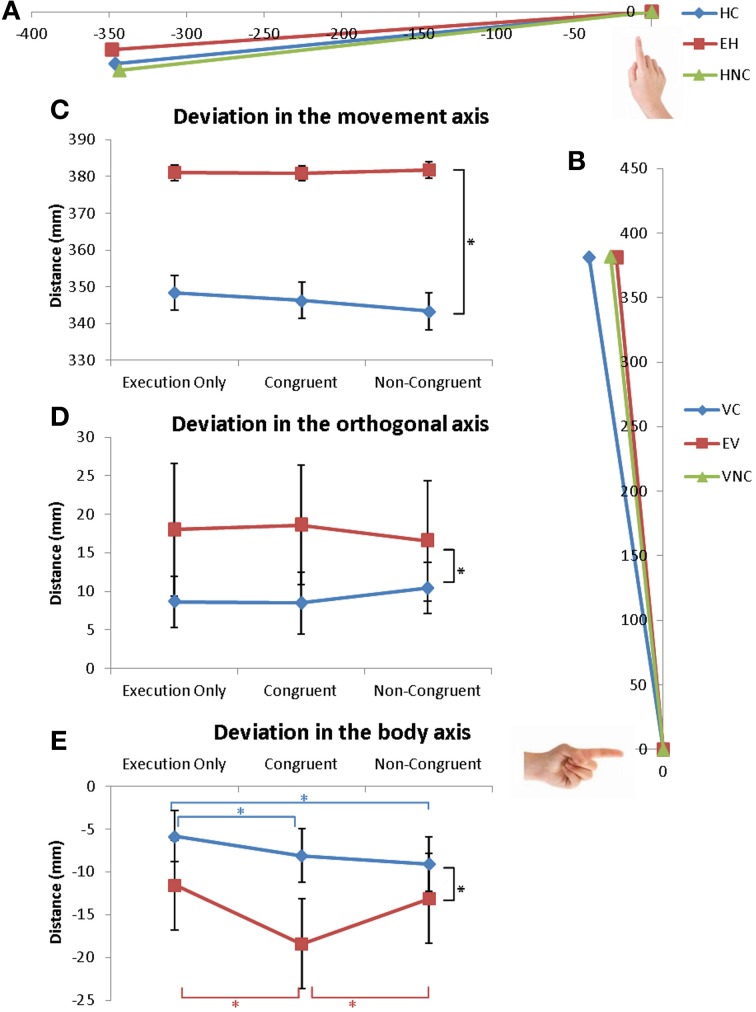
**Coordinates of the extrema of the trajectory. (A,B)** Extreme position of the finger during **(A)** horizontal and **(B)** vertical movements for the 3 conditions. **(C–E)** Mean values of the extreme position of the trajectory in the **(C)** movement axis, **(D)** in the orthogonal axis and **(E)** in the body axis. Error bars represent the standard error of the mean (s.e.m.). ^*^p = 0.05. Dark lines correspond to a main effect of condition whereas colored lines correspond to specific effects of horizontal (red) or vertical (blue) movements.

The same analyses were conducted for the observer and are reported in Figures 2, 3 and Table 1.

**Table 1:** **Observer's Averaged values for all analyzed parameters for each condition [Mean values ± standard error of the mean (s.e.m.)]**.

**Conditions**		**Total**	**1st part of movement**			**2nd part of movement**			**Deviation of the extrema of the trajectory (mm)**				
		**Duration *t* (ms)**	**Vel_1_ (mm/s)**	**LatPeak_1_ (ms)**	**Duration_1_ (ms)**	**Vel_2_ (mm/s)**	**LatPeak_2_ (ms)**	**Duration_2_ (ms)**	**Movement deviation**	**Orthogonal deviation**	**Body deviation**	**Reaction time (ms)**	**DMA—DMO (ms)**
Horizontal	Movement only	2018 ± 102	681 ± 46	405 ± 22	908 ± 45	615 ± 39	465 ± 26	1114 ± 59	348 ± 5	9 ± 2	−6 ± 3	217 ± 24	
	Congruent	1946 ± 94	738 ± 45	388 ± 20	853 ± 46	622 ± 40	452 ± 22	1087 ± 53	346 ± 5	8 ± 2	−8 ± 3	192 ± 17	231 ± 22
	Non-congruent	1974 ± 94	716 ± 43	388 ± 19	873 ± 42	615 ± 38	448 ± 20	1103 ± 55	343 ± 5	10 ± 2	−9 ± 3	195 ± 19	233 ± 25
Vertical	Movement only	2016 ± 94	837 ± 53	404 ± 25	877 ± 43	682 ± 44	431 ± 25	1139 ± 54	381 ± 8	17 ± 3	−12 ± 5	230 ± 23	
	Congruent	1892 ± 96	896 ± 53	365 ± 18	812 ± 39	719 ± 45	431 ± 23	1082 ± 59	381 ± 8	18 ± 3	−18 ± 5	194 ± 17	229 ± 23
	Non-congruent	1961 ± 98	872 ± 54	381 ± 20	839 ± 40	697 ± 43	432 ± 23	1126 ± 61	382 ± 8	17 ± 3	−13 ± 5	200 ± 20	242 ± 28
ANOVA (Direction)		*F*_(1, 16)_ = 1.11; ns	*F*_(1, 16)_ = 45.32; *p* < 0.0001	*F*_(1, 16)_ = 2.88; ns	*F*_(1, 16)_ = 9.05; *p* < 0.01	*F*_(1, 16)_ = 44.22; *p* < 0.0001	*F*_(1, 16)_ = 10.21; *p* < 0.06	*F*_(1, 16)_ = 0.63; ns	*F*_(1, 16)_ = 43.9; *p* < 0.0001	*F*_(1, 16)_ = 4.4; *p* = 0.05	*F*_(1, 16)_ = 2.45; ns	*F*_(1, 16)_ = 0.60; ns	*F*_(1, 16)_ = 0.16; ns
ANOVA (Condition)		*F*_(2, 32)_ = 23.58; *p* < 0.0001	*F*_(2, 32)_ = 14.66; *p* < 0.0001	*F*_(2, 32)_ = 2.83; *p* < 0.01	*F*_(2, 32)_ = 19.66; *p* < 0.0001	*F*_(2, 32)_ = 18.45; *p* < 0.0001	*F*_(2, 32)_ = 0.57; ns	*F*_(2, 32)_ = 9.42; *p* < 0.001	*F*_(2, 32)_ = 1.3; ns	*F*_(2, 32)_ = 0.1; ns	*F*_(2, 32)_ = 21; *p* < 0.0001	*F*_(2, 32)_ = 3.92; *p* < 0.03	*F*_(1, 16)_ = 0.62; ns
ANOVA (Direction * Condition)		*F*_(2, 32)_ = 3.75; *p* < 0.035	*F*_(2, 32)_ = 0.01; ns	*F*_(2, 32)_ = 3.65; *p* < 0.05	*F*_(2, 32)_ = 0.61; ns	*F*_(2, 32)_ = 4.42; *p* < 0.03	*F*_(2, 32)_ = 1.60; ns	*F*_(2, 32)_ = 1.99; ns	*F*_(2, 32)_ = 2.9; ns	*F*_(2, 32)_ = 2.5; ns	*F*_(2, 32)_ = 13.65; *p* < 0.0001	*F*_(2, 32)_ = 0.63; ns	*F*_(1, 16)_ = 0.56; ns

#### Movement onset

The observer's RT was shorter when the actor was performing a movement. ANOVA revealed a significant effect of Condition [*F*_(2, 32)_ = 3.92; *p* < 0.03]. In the Execution only condition, RT was delayed compared to RT in the Congruent condition (*p* < 0.04) and in the Non-Congruent condition (*p* < 0.04) (see Figure 2B). No differences were found between the Congruent and the Non-Congruent conditions. Similarly, the delay between the onset of the observer's movement and the onset of the actor's movement was not different between the Congruent and Non-Congruent conditions (ANOVA Condition: *F*_(1, 16)_ = 0.62; ns).

#### Total movement duration

A repeated-measures ANOVA (Direction × Condition) revealed a significant effect of the Direction * Condition interaction [*F*_(2, 32)_ = 3.75; *p* < 0.035]. On average, the total duration of movement was longer during the Execution only condition (EH and EV, respectively) than during the Congruent (*p* < 0.0005, *p* < 0.0002) and the Non-Congruent trials (*p* < 0.02 and *p* < 0.002). Moreover, movements in the VC condition were also shorter than in the VNC condition (*p* < 0.0002) (see Figure 2A). However, no difference was observed between HC and HNC conditions.

#### First sub-phase of the movement

Movement duration and velocity peak amplitude were affected by the observation of the actor's movement. An ANOVA revealed a significant effect of Direction [Duration_1_: *F*_(1, 16)_ = 9.05; *p* < 0.01 and Vel_1_: *F*_(1, 16)_ = 45.32; *p* < 0.0001] and a significant effect of Condition [Duration_1_: *F*_(2, 32)_ = 19.66; *p* < 0.0001, Vel_1_: *F*_(2, 32)_ = 14.66; *p* < 0.0001]. *Post-hoc* analyses showed that movements were shorter and faster in the Congruent condition than in the Execution only (Duration_1_: *p* < 0.0002; Vel_1_: *p* < 0.0002) and the Non-Congruent condition (Duration_1_: *p* < 0.03; Vel_1_: *p* < 0.02). Additionally, the movements in the Non-Congruent condition were faster than the condition Execution only (Duration_1_: *p* < 0.001; Vel_1_: *p* < 0.004). The results are displayed Figures 2C,E.

Concerning the velocity peak latencies, the ANOVA revealed a significant interaction of Direction * Condition [*F*_(2, 32)_ = 3.65; *p* < 0.04]. *Post-hoc* analyses revealed that the velocity peak was more delayed in the Execution only condition (EH and EV, respectively) than in the Congruent (*p* < 0.03, *p* < 0.0002) and the Non-Congruent trials (*p* < 0.04 and *p* < 0.004) (see Figure 2G). Moreover, velocity peaks in the VC condition occurred earlier than in the VNC condition (*p* < 0.02). However, no differences were observed between the HC and HNC conditions.

#### Second sub-phase of the movement

Movement duration and velocity peak amplitude were also affected by the observation of the actor's movement. For Duration_2_, a repeated-measures ANOVA revealed an effect of Condition [*F*_(2, 32)_ = 9.42; *p* < 0.001]. *Post-hoc* analyses showed that movements were shorter in the Congruent condition than in the Execution only (*p* < 0.001) and Non-Congruent conditions (*p* < 0.006) as illustrated Figure 2D.

An ANOVA for the Velocity peak amplitude (Vel_2_) revealed an effect of Direction [*F*_(1, 16)_ = 44.22; *p* < 0.0001], Condition [*F*_(2, 32)_ = 18.45; *p* < 0.0001], and an interaction of Direction * Condition [*F*_(2, 32)_ = 4.42; *p* < 0.03]. *Post-hoc* analyses revealed that the Vertical movements were faster than the Horizontal movements. Moreover, movements were faster in the VC condition than in the EV (*p* < 0.0002) and VNC (*p* < 0.005) conditions. Additionally, Vel_2_ in the VNC condition was also stronger than in the EV condition (*p* < 0.05). No differences were observed for horizontal movements (see Figure2F).

Concerning the velocity peak latencies (Lat_2_), the ANOVA revealed a significant effect of Direction [*F*_(1, 16)_ = 10.21; *p* < 0.006] but no effect of Condition [*F*_(2, 32)_ = 0.57; ns] (see Figure 2H).

#### Coordinate of the extrema of the trajectory

The results are reported Figure 3. Deviations in the three directions were analyzed: deviation in the movement direction (X-axis for horizontal and Z-axis for vertical movements), deviation in the orthogonal axis to movement direction (Z-axis for horizontal and X-axis for vertical movements) and deviation from the body (Y-axis) (Figures 3A,B).

No effects of Condition were observed for the deviation in movement direction or in the orthogonal direction [*F*_(2, 32)_ = 1.3; ns and *F*_(2, 32)_ = 0.947; ns] (Figures 3C,D).

Coordinates over the y-axis revealed no effect of Direction [*F*_(1, 16)_ = 2.45; ns] but the interaction of Direction * Condition was significant [*F*_(2, 32)_ = 13.7; *p* < 0.0001]. For vertical movements, movements in the VC condition were different from movements in the EV (*p* < 0.0002) and VNC conditions (*p* < 0.0002). No difference was measured between the VNC and EV conditions as illustrated in the Figures 3B,E.

For horizontal movements, movements in the HC and HNC conditions were different from movements in the EH condition (*p* < 0.04 and *p* < 0.001) but were not different from each other (see Figures 3A,E). For both directions, the trajectories were more directed toward the participants (more curved) in the VC, HC and HNC conditions compared to the control conditions (EV and EH). Here also, greater modulation was seen in vertical movements than in horizontal.

In conclusion, movement observation affected multiple parameters of movement execution. RT was shorter and movements were faster and shorter when observing any movement by the actor (Congruent or Non-Congruent). However, movements were even more accelerated when observing congruent movements rather than Non-Congruent movements (Dur_1_, Vel_1_, Dur_2_). Vertical movements seemed globally more affected by the congruency of the observed action than horizontal movements (Dur_*t*_, Vel_2_, Lat_1_, Y-axis). Moreover, the movement's trajectory was also more curved when observing an action over the y-axis.

### Electrophysiological results

**Figure 4:**
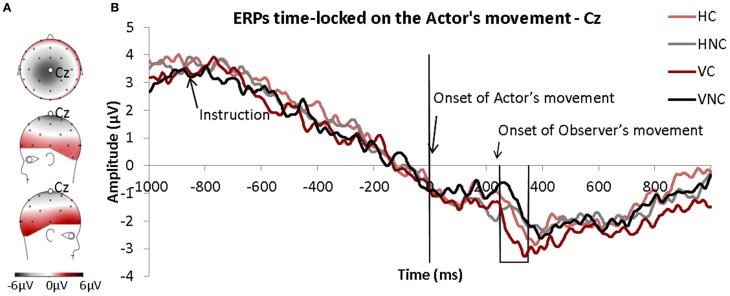
**(A)** Topography of the motor related potentials (MRP) averaged for all conditions and time-locked to the onset of the observers' movements. **(B)** ERPs time-locked to the onset of actors' movements (time 0) for each condition. The frame corresponds to the window of interest.

Figure 4 represents the observer's ERPs time-locked to the onset of the actor's movements measured over the Cz electrode. The Cz electrode was chosen because the MRP such as the Readiness Potential and the CNV are commonly described (Libet, 1985) over this electrode and, more specifically, because it was only observed over this electrode in our experiment (see Figure 4A). Because we wanted to determine the influence of motor observation on motor behavior, the ERPs were time-locked to the onset of the actor's movements in spite of the “Go” signal to avoid time variability effects in the actors' RT during the experiment. Therefore, the onset of the observer's movements occurred on average 234 ms after the onset of the actor's movements (time 0).

A MRP was clearly observed in all conditions. It was present as early as 800 ms before the onset of the actor's movements. The MRP was comparable between all conditions except during a short time-window from 250 ms to 350 ms after the onset of the actor's movements (preliminary *t*-test analysis). The repeated-measures ANOVA (Direction × Condition) performed for this 100 ms time window (250–350 ms) revealed a significant effect of Condition. The MRP was significantly more negative in the Congruent condition than in the Non-Congruent condition (see Figure 4B).

## Discussion

Our goal was to determine whether the simultaneous activation of the MNS/motor areas during the observation and the execution of an action influenced movement execution and whether this influence was discernable in the EEG. Moreover, through the coupling of kinematics and EEG recordings, we further sought to identify the optimal time-window for inducing this facilitation. Fine-grained analysis of the kinematics of the movement and the observer's brain activity revealed that the observation of a congruent movement facilitated the execution of a movement, and that within a short time-window. This facilitation effect was discernable in the brain correlates of the motor activity. Indeed, we found that the movements in the Congruent condition were faster (i.e., velocity) and shorter (i.e., duration) than in the Execution only and in the Non-Congruent conditions. The MRPs synchronized to the onset of the actor's movements also seemed to be briefly influenced by the congruency of the movement observed. MRPs were more negative in the Congruent condition compared to the Non-Congruent condition approximately 300 ms after the onset of the actor's movements.

### Movement observation facilitates congruent movement execution

Regarding the kinematics of the movement, our main finding was that the observation of an action, particularly a congruent action, increased the speed of movement. Indeed, compared to the Execution only and the Non-Congruent conditions, movements in the congruent condition exhibited larger and earlier velocity peaks (i.e., Vel_1/2_, Lat_1_) and shorter durations (i.e., Duration 1,2 *t*). Movement trajectories were also slightly deflected by the observation of an action, particularly in the Congruent condition (i.e., movements in the Y-axis). It is known that non-constrained vertical arm movement exhibits a curved trajectory (Atkeson and Hollerbach, 1985; Wada et al., 2006). Therefore, the greater curvatures of the trajectories observed in the Congruent movement may have resulted from the modification of elbow/shoulder flexion to optimize movement execution (Alexander, 1997; Wada et al., 2006) or to increase movement velocity (Van Thiel et al., 1998). All of these modulations seem to reflect facilitations of the actions in the Congruent condition and were measured over multiple kinematic parameters confirming their robustness. Such findings are consistent with previous behavioral studies (Brass et al., 2000; Ménoret et al., 2013); we measured similar effects during the execution of grasping actions in our previous experiment (Ménoret et al., 2013).

The modulations of movement parameters reported here were stronger for vertical movements than for horizontal movements. This differential influence of movement direction has also been reported by Stanley et al. (2007) and may be related to visual field properties. In our study, participants were asked to fixate on a point and observe the movement with peripheral vision. Carrasco and others have shown that performance in visual search tasks is optimal when the target observed is on the horizontal meridian (Carrasco and Frieder, 1997; Mackeben, 1999; Carrasco et al., 2004). Therefore, this “horizontal–vertical anisotropy” suggests that the observation of horizontal movement during the execution of a vertical movement may be more salient than the observation of a vertical movement during a horizontal movement and may explain this differential influence.

Contrary to Brass et al. (2000) and Kilner et al. (2003), we did not observe any interference effects during the observation of a non-congruent movement. Note though that by coupling movement analysis with EEG we were constrained to modify our experimental paradigm. Hence, in contrast to previous studies, in our paradigm, the observer received the instructions for the to-be executed movement before observing the actor's action. The analysis of the observer's ERPs, synchronized to the actor's movement onset, displayed an MRP up to 800 ms before the onset of the actor's movement. This observation indicates that the observer was already preparing his/her movement when the actor's movement occurred. Hence, the influence of movement observation occurred during action preparation and not during movement decision (Brass et al., 2000; Poljac et al., 2009; Ocampo and Kritikos, 2010).

The present experiment also differed significantly from Kilner et al.'s initial study (2003), specifically regarding the type of movement performed and the delay between the observed and executed actions. In that study, participants had to perform synchronous continuous actions (lasting approximately 20 s), whereas in our experiment, participants performed discrete actions (lasting 2 s). Continuous and long actions are more susceptible to variance than discrete actions. Additionally, due to the constraints of the EEG, our subjects were instructed to not move their eyes, and all trials containing errors (e.g., correction of the movement's trajectory during execution) were removed from analysis to avoid contamination of the EEG data. Taken together, these differences may have masked any potential interference effects. Alternate analyses, i.e., a continuous analysis, could help identify interfering effects including those related to partial errors, as identified in McBride et al.'s study for instance (McBride et al., 2012).

### Functional interpretation: insights from the MRPs

The analyses of electrophysiological motor potentials provided several clues for understanding the time-course of motor facilitation in this paradigm. The MRPs were quite similar between conditions except in the 250–350 ms time window following the onset of the actor's movement. In this time period, a significant difference between the Congruent and Non-Congruent trials was found; the MRP was more negative in the Congruent condition compared to the Non-Congruent condition. Additionally, the effect was stronger for vertical movements than for horizontal movements, and this finding was consistent with the kinematic data. Given the localization of the effect and that it was restricted to the time-window around the onset of the observer's movement, the variation measured on the MRPs seems to be related to the motor execution of the action. The MRPs were synchronized to the onset of the observed movement, indicating that this effect was directly related to the observation of the actor. One can assume that the neuronal mechanisms underlying this facilitation are related to the mirror neurons system.

According to the hypothesis of mirror neurons, the observation of an action induces an automatic activation of the MNS that involves the STS, parietal and motor areas and possibly mirror neurons in other areas (Mukamel et al., 2010). Alternately, this effect could also rely on purely spatial and non-motor related facilitation. Indeed, as our experiment did not contain non-biological/non-motor conditions, these effects may have been produced by the observation of the visual stimuli alone and may be related only to visual information (Press et al., 2006; Stanley et al., 2007; Tsai et al., 2008; Dolk et al., 2011, 2013). For example, in a single person experiment, Dolk et al. (2011, 2013) reported a “joint *social* Simon effect” in the presence of only a salient visual stimulus. These authors interpreted this effect within the framework of the Theory of Event Coding and the ideomotor theory (Hommel, 2009); thus, actions observed, even actions performed by an object, could be coded in terms of sensory consequences. From the perspective of these authors, it is therefore, possible that the visual stimulation induced activation of the sensory system (and potentially the MNS) even though this stimulation was not performed by a human agent (Press et al., 2006; Tsai et al., 2008).

This activation of the sensory-motor system prior to motor execution may prime the motor system and facilitate motor initiation and motor execution, resulting in the observed facilitation of the concurrent motor response. These effects are in accordance with other studies that have investigated the activation of motor areas during language processing. Indeed, it has been shown that not only observing actions but also perceiving or pronouncing action words can activate the motor system (Tettamanti et al., 2005; Aziz-Zadeh and Damasio, 2008; Boulenger et al., 2009; Pulvermüller and Fadiga, 2010; Fargier et al., 2012). Aravena et al. (2010), for instance, studied whether the compatibility of a performed hand action and an action described in a sentence would influence brain motor potentials. The authors showed that participants' manual responses were accelerated when the performed action and the action described in the sentence were compatible. Moreover, the amplitudes of motor potentials and reafferent potentials were larger in the compatible condition, indicating that motor responses are facilitated when language and motor processes are congruent. These results are in accordance with the observations of the present study and provide evidence that the co-activation of motor areas can facilitate movement execution within an restricted time-window.

Alternatively, the difference between the Congruent and Non-congruent conditions may also have resulted from an inhibition of the imitative NC response. Indeed, the absence of EEG data for the Execution Only condition (these data could not be synchronized to the actor's movement because no movement was performed) prevents us from ruling out this hypothesis, which was developed by Brass and colleagues. In their fMRI studies, Brass et al. (2001b) and Stanley and Miall (2007) found that non-congruent movement observation induces stronger activity in the parietal/prefrontal and premotor areas, and this finding is suggestive of this type of inhibition. Moreover, in a Go/No-Go task, Sebanz et al. (2006) reported stronger positivities around 300 ms after the No-Go onset when participants were suppressing an action and acting with a co-actor compared to when they were acting alone. This results confirms that the inhibition of action is stronger in social contexts (Sebanz et al., 2006). According to Barchiesi and Cattaneo (2013), the period of approximately 300 ms after the onset of an action may represent a crucial period in which the motor system suppresses an automatic imitative tendency. In a learning TMS experiment, these authors measured a dissociation in motor processing approximately between 250 and 300 ms and argued that this dissociation could underlie two different mechanisms of motor processing: early processing (i.e., within the first 250 ms), which represents an automatic “mirror-like” process that is not affected by short-term learning, and a second mechanism after 300 ms that may depend on newly learned associations.

These results indicate that inhibitions of imitative behavior may have occurred around 300 ms after the onset of the actor's movement in the present study, although we did not measure any behavioral interference effect in the non-congruent condition. The absence of an interference effect implies that this inhibitory process takes place as reported by Brass but did not impact kinematics in the present experiment. It is also possible that both a facilitation of congruent action and an inhibition of the non-congruent movement co-occur and are triggered by different neural processes.

### Non-specific facilitation: a competition effect?

Similar to our previous experiment (Ménoret et al., 2013), here we reported a non-specific facilitating effect of movement observation. Hence, compared to the Execution only condition, the observers' movements were faster (e.g., RT, duration and velocity) when observing an action, independently of whether this action was congruent with his/her own movement or not (Congruent or Non-Congruent). However, the facilitation was larger for congruent movements than for incongruent movements. This non-specific effect has previously been observed in a similar paradigm (Ménoret et al., 2013), and it is likely that it results from competitive behavior. Indeed, because the actors started their movements prior to the observers, the observers may have attempted to catch up with the actor to perform the movement simultaneously. Within the framework of the mirror neuron hypothesis, we may also imagine that MNS activation may also be responsible for the non-specific facilitation that was independent of the congruency of the movement to be executed because of the intrinsic properties of mirror neurons. In monkeys, specifically congruent neurons and broadly congruent neurons have been described, and these populations exist in a one-third to two-thirds proportion, respectively, (Rizzolatti and Craighero, 2004). This finding implies that the observation of two different actions with a common goal activates a common group of mirror neurons. A similar pattern likely exists in humans for both goal-directed and aimless movements (Newman-Norlund et al., 2007). Therefore, we hypothesize that a number of the mirror neurons that are activated during the observation of the same action are also activated during the observation of non-congruent movements. These neurons may be responsible for the gradual facilitation we observed in the present experiment in both the Congruent and Non-Congruent conditions. This activation of the MNS that occurs before movement onset may prime the motor system independently of the direction of the movement and result in faster movement initiation times, as revealed by RT.

### The temporal tuning of the co-activation of the motor system

Although this study did not resolve the issue of the facilitatory or inhibitory effect on motor activity, its main contribution lies in the illumination of the temporal dynamics of the modulation of brain activity. We have shown that the MRPs only differed between congruent and non-congruent conditions within a short time window around 300 ms after the onset of the actor's movement. On average, this time window coincided with the beginning of the observers' actions because no differences were measured between the onsets of the observer's movements in these two conditions. According to Babiloni et al. (2003), this time window also corresponds to the processing of observed aimless movements. Indeed, these authors reported that observing aimless movements induces a rapid cortical response lasting approximately 200–400 ms after movement onset, as measured via the N200 and P300 potentials over the parietal areas. Therefore, the effects on the MRPs measured in our experiment occurred both when the observer processed the observed movement and when the observer initiated his/her own movement. This synchrony should result in optimal facilitation because motor execution occurred during the optimal time window for facilitating concurrent movement execution. This hypothesis is supported by Christensen et al. (2011); Catmur et al. (2011) and our previous work that showed that facilitation disappeared if the interval between observation and execution events was excessive (Ménoret et al., 2013). Therefore, this temporal tuning seems to be essential for the influence of motor execution influence. Thus, the variations in temporal tuning across the different protocols may account for the behavioral and neuronal discrepancies reported in previous studies (facilitation vs. interference) (Brass et al., 2000; Poljac et al., 2009; Ocampo and Kritikos, 2010) and should be taken into account in future studies.

These findings may also produce new insights in joint action research. Indeed, tight coordination is essential to achieve common goals, for example, carrying heavy objects (Sebanz et al., 2006). The transient effect measured within a tight time-window may explain how such temporal coordination arises, i.e., through the optimization of motor perception and motor execution. This interpretation may explain the importance of coordination in successful joint action (Richardson and Dale, 2005; Knoblich et al., 2011).

## Conclusions

The coupling of kinematics and electrophysiological recordings that were synchronized to the onset of the movements allowed us to precisely monitor brain motor activity and motor responses during movement observation and execution. We documented the facilitation of executed actions during the observation of congruent actions that induced a modified MRP. This effect is likely related to the pre-activation of the MNS or from a more visual/spatial compatibility effect. This facilitation seemed to depend not only on spatial congruency but also, and to a greater extent, on the optimal temporal relationship of the observation and execution events. For further research, it is essential to take into account not only movement congruency but also the temporal tuning of the observed/executed actions to draw conclusions about this effect. Finally, the coupling between kinematics and EEG recordings provided temporal insights into variations in kinematics. Therefore, this technique should provide interesting information in further studies of real interactions between participants.

### Conflict of interest statement

The authors declare that the research was conducted in the absence of any commercial or financial relationships that could be construed as a potential conflict of interest.

## Supplementary material

Supplementary Table 1 **Actor's Averaged values for all analyzed parameters for each condition [Mean values ± standard error of the mean (s.e.m.)]**.

## References

[B1] Alexander R. M. (1997). A minimum energy cost hypothesis for human arm trajectories. Biol. Cybern. 76, 97–105 10.1007/s004220050324 9116080

[B2] Aravena P. Hurtado E. Riveros R. Cardona J. F. Manes F. Ibáñez A. (2010). Applauding with closed hands: neural signature of action-sentence compatibility effects. PLoS ONE 5:e11751 10.1371/journal.pone.0011751 20676367 PMC2911376

[B3] Atkeson C. G. Hollerbach J. M. (1985). Kinematic features of unrestrained vertical arm movements. J. Neurosci. 5, 2318–2330 4031998 10.1523/JNEUROSCI.05-09-02318.1985 PMC6565321

[B4] Aziz-Zadeh L. Damasio A. (2008). Embodied semantics for actions: findings from functional brain imaging. J. Physiol. Paris 102, 35–39 10.1016/j.jphysparis.2008.03.012 18472250

[B5] Babiloni C. Del Percio C. Babiloni F. Carducci F. Cincotti F. Moretti D. V. (2003). Transient human cortical responses during the observation of simple finger movements: a high-resolution EEG study. Hum. Brain Mapp. 20, 148–157 10.1002/hbm.10135 14601141 PMC6872072

[B6] Barchiesi G. Cattaneo L. (2013). Early and late motor responses to action observation. Soc. Cogn. Affect Neurosci. 8, 711–719 10.1093/scan/nss049 22563004 PMC3739914

[B7] Barchiesi G. Wache S. Cattaneo L. (2012). The frames of reference of the motor-visual aftereffect. PLoS ONE 7:e40892 10.1371/journal.pone.0040892 22848406 PMC3406615

[B8] Blakemore S.-J. Frith C. (2005). The role of motor contagion in the prediction of action. Neuropsychologia 43, 260–267 10.1016/j.neuropsychologia.2004.11.012 15707910

[B9] Boulenger V. Hauk O. Pulvermüller F. (2009). Grasping ideas with the motor system: semantic somatotopy in idiom comprehension. Cereb. Cortex 19, 1905–1914 10.1093/cercor/bhn217 19068489 PMC2705699

[B11] Brass M. Bekkering H. Wohlschläger A. Prinz W. (2000). Compatibility between observed and executed finger movements: comparing symbolic, spatial, and imitative cues. Brain Cogn. 44, 124–143 10.1006/brcg.2000.1225 11041986

[B10] Brass M. Bekkering H. Prinz W. (2001a). Movement observation affects movement execution in a simple response task. Acta Psychol. (Amst) 106, 3–22 10.1016/S0001-6918(00)00024-X 11256338

[B12] Brass M. Zysset S. von Cramon D. Y. (2001b). The inhibition of imitative response tendencies. Neuroimage 14, 1416–1423 10.1006/nimg.2001.0944 11707097

[B13] Buccino G. Binkofski F. Fink G. R. Fadiga L. Fogassi L. Gallese V. (2001). Action observation activates premotor and parietal areas in a somatotopic manner: an fMRI study. Eur. J. Neurosci. 13, 400–404 10.1111/j.1460-9568.2001.01385.x 11168545

[B14] Carrasco M. Frieder K. S. (1997). Cortical magnification neutralizes the eccentricity effect in visual search. Vision Res. 37, 63–82 10.1016/S0042-6989(96)00102-2 9068831

[B15] Carrasco M. Marie Giordano A. McElree B. (2004). Temporal performance fields: visual and attentional factors. Vision Res. 44, 1351–1365 10.1016/j.visres.2003.11.026 15066395

[B16] Casile A. Giese M. A. (2006). Nonvisual motor training influences biological motion perception. Curr. Biol. 16, 69–74 10.1016/j.cub.2005.10.071 16401424

[B17] Catmur C. Mars R. B. Rushworth M. F. Heyes C. (2011). Making mirrors: premotor cortex stimulation enhances mirror and counter-mirror motor facilitation. J. Cogn. Neurosci. 23, 2352–2362 10.1162/jocn.2010.21590 20946056

[B18] Cattaneo L. Barchiesi G. Tabarelli D. Arfeller C. Sato M. Glenberg A. M. (2011). One's motor performance predictably modulates the understanding of others' actions through adaptation of premotor visuo-motor neurons. Soc. Cogn. Affect. Neurosci. 6, 301–310 10.1093/scan/nsq099 21186167 PMC3110437

[B19] Christensen A. Ilg W. Giese M. A. (2011). Spatiotemporal tuning of the facilitation of biological motion perception by concurrent motor execution. J. Neurosci. 31, 3493–3499 10.1523/JNEUROSCI.4277-10.2011 21368061 PMC6623932

[B20] Di Pellegrino G. Fadiga L. Fogassi L. Gallese V. Rizzolatti G. (1992). Understanding motor events: a neurophysiological study. Exp. Brain Res. 91, 176–180 10.1007/BF00230027 1301372

[B21] Dolk T. Hommel B. Colzato L. S. Schütz-Bosbach S. Prinz W. Liepelt R. (2011). How “social” is the social Simon effect? Front. Psychol. 2:84 10.3389/fpsyg.2011.00084 21687453 PMC3110342

[B22] Dolk T. Hommel B. Prinz W. Liepelt R. (2013). The (Not So) social simon effect: a referential coding account. J. Exp. Psychol. Hum. Percept. Perform. [Epub ahead of print]. 10.1037/a0031031 23339346

[B23] Fagioli S. Hommel B. Schubotz R. I. (2007). Intentional control of attention: action planning primes action-related stimulus dimensions. Psychol. Res. 71, 22–29 10.1007/s00426-005-0033-3 16317565

[B24] Fargier R. Ménoret M. Boulenger V. Nazir T. A. Paulignan Y. (2012). Grasp it loudly! Supporting actions with semantically congruent spoken action words. PLoS ONE 7:e30663 10.1371/journal.pone.0030663 22292014 PMC3265503

[B25] Gallese V. Fadiga L. Fogassi L. Rizzolatti G. (1996). Action recognition in the premotor cortex. Brain 119(Pt 2), 593–609 10.1093/brain/119.2.593 8800951

[B26] Gallese V. Goldman A. (1998). Mirror neurons and the simulation theory of mind-reading. Trends Cogn. Sci. (Regul. Ed.) 2, 493–501 10.1016/S1364-6613(98)01262-5 21227300

[B27] Hommel B. (2009). Action control according to TEC (theory of event coding). Psychol. Res. 73, 512–526 10.1007/s00426-009-0234-2 19337749 PMC2694931

[B28] Jeannerod M. (2001). Neural simulation of action: a unifying mechanism for motor cognition. Neuroimage 14, S103–S109 10.1006/nimg.2001.0832 11373140

[B29] Keysers C. Gazzola V. (2010). Social neuroscience: mirror neurons recorded in humans. Curr. Biol. 20, R353–R354 10.1016/j.cub.2010.03.013 21749952

[B30] Kilner J. M. Marchant J. L. Frith C. D. (2009). Relationship between activity in human primary motor cortex during action observation and the mirror neuron system. PLoS ONE 4:e4925 10.1371/journal.pone.0004925 19290052 PMC2654140

[B31] Kilner J. M. Paulignan Y. Blakemore S. J. (2003). An interference effect of observed biological movement on action. Curr. Biol 13, 522–525 10.1016/S0960-9822(03)00165-9 12646137

[B32] Knoblich G. Butterfill S. Sebanz N. (2011). 3 Psychological research on joint action: theory and data. Psychol. Learn. Motiv. Adv. Res. Theor. 54, 59 10.1016/B978-0-12-385527-5.00003-6

[B33] Leuthold H. Sommer W. Ulrich R. (2004). Preparing for action: inferences from CNV and LRP. J. Psychophysiol. 18, 77–88 10.1027/0269-8803.18.23.77

[B34] Libet B. (1985). Unconscious cerebral initiative and the role of conscious will in voluntary action. Behav. Brain Sci. 8, 529–539 10.1017/S0140525X00044903

[B35] Mackeben M. (1999). Sustained focal attention and peripheral letter recognition. Spat. Vis. 12, 51–72 10.1163/156856899X00030 10195388

[B36] McBride J. Boy F. Husain M. Sumner P. (2012). Automatic motor activation in the executive control of action. Front. Hum. Neurosci. 6:82 10.3389/fnhum.2012.00082 22536177 PMC3334842

[B37] Ménoret M. Curie A. des Portes V. Nazir T. A. Paulignan Y. (2013). Simultaneous action execution and observation optimise grasping actions. Exp. Brain Res. 227, 407–419 10.1007/s00221-013-3523-3 23615976

[B38] Miall R. C. Stanley J. Todhunter S. Levick C. Lindo S. Miall J. D. (2006). Performing hand actions assists the visual discrimination of similar hand postures. Neuropsychologia 44, 966–976 10.1016/j.neuropsychologia.2005.09.006 16249009

[B39] Mukamel R. Ekstrom A. D. Kaplan J. Iacoboni M. Fried I. (2010). Single-Neuron responses in humans during execution and observation of actions. Curr. Biol. 20, 750–756 10.1016/j.cub.2010.02.045 20381353 PMC2904852

[B40] Newman-Norlund R. D. van Schie H. T. van Zuijlen A. M. J. Bekkering H. (2007). The mirror neuron system is more active during complementary compared with imitative action. Nat. Neurosci. 10, 817–818 10.1038/nn1911 17529986

[B41] Ocampo B. Kritikos A. (2010). Placing actions in context: motor facilitation following observation of identical and non-identical manual acts. Exp. Brain Res. 201, 743–751 10.1007/s00221-009-2089-6 19937320

[B42] Oldfield R. C. (1971). The assessment and analysis of handedness: the Edinburgh inventory. Neuropsychologia 9, 97–113 10.1016/0028-3932(71)90067-4 5146491

[B43] Pineda J. A. (2008). Sensorimotor cortex as a critical component of an ‘extended’mirror neuron system: does it solve the development, correspondence, and control problems in mirroring? Behav. Brain Funct. 4:47 10.1186/1744-9081-4-47 18928566 PMC2577683

[B44] Poljac E. van Schie H. T. Bekkering H. (2009). Understanding the flexibility of action-perception coupling. Psychol. Res. 73, 578–586 10.1007/s00426-009-0238-y 19347358 PMC2694934

[B45] Press C. Gillmeister H. Heyes C. (2006). Bottom-up, not top-down, modulation of imitation by human and robotic models. Eur. J. Neurosci. 24, 2415–2419 10.1111/j.1460-9568.2006.05115.x 17042792

[B46] Prinz W. (1997). Perception and action planning. Eur. J. Cogn. Psychol. 9, 129–154 10.1080/713752551

[B47] Pulvermüller F. Fadiga L. (2010). Active perception: sensorimotor circuits as a cortical basis for language. Nat. Rev. Neurosci. 11, 351–360 10.1038/nrn2811 20383203

[B48] Ramsey R. Cumming J. Eastough D. Edwards M. G. (2010). Incongruent imagery interferes with action initiation. Brain Cogn. 74, 249–254 10.1016/j.bandc.2010.08.005 20846772

[B49] Richardson D. C. Dale R. (2005). Looking to understand: the coupling between speakers' and listeners' eye movements and its relationship to discourse comprehension. Cogn. Sci. 29, 1045–1060 10.1207/s15516709cog0000_29 21702802

[B50] Rizzolatti G. Craighero L. (2004). The mirror-neuron system. Annu. Rev. Neurosci. 27, 169–192 10.1146/annurev.neuro.27.070203.144230 15217330

[B51] Rizzolatti G. Fogassi L. Gallese V. (2001). Neurophysiological mechanisms underlying the understanding and imitation of action. Nat. Rev. Neurosci. 2, 661–670 10.1038/35090060 11533734

[B52] Schütz-Bosbach S. Prinz W. (2007). Perceptual resonance: action-induced modulation of perception. Trends Cogn. Sci. (Regul. Ed.) 11, 349–355 10.1016/j.tics.2007.06.005 17629544

[B53] Sebanz N. Bekkering H. Knoblich G. (2006). Joint action: bodies and minds moving together. Trends Cogn. Sci. (Regul. Ed.) 10, 70–76 10.1016/j.tics.2005.12.009 16406326

[B54] Stanley J. Gowen E. Miall R. C. (2007). Effects of agency on movement interference during observation of a moving dot stimulus. J. Exp. Psychol. Hum. Percept. Perform. 33, 915–926 10.1037/0096-1523.33.4.915 17683237 PMC3073012

[B55] Stanley J. Miall R. C. (2007). Functional activation in parieto-premotor and visual areas dependent on congruency between hand movement and visual stimuli during motor-visual priming. Neuroimage 34, 290–299 10.1016/j.neuroimage.2006.08.043 17056279

[B56] Tettamanti M. Buccino G. Saccuman M. C. Gallese V. Danna M. Scifo P. (2005). Listening to action-related sentences activates fronto-parietal motor circuits. J. Cogn. Neurosci. 17, 273–281 10.1162/0898929053124965 15811239

[B57] Tsai C.-C. Kuo W.-J. Hung D. L. Tzeng O. J. L. (2008). Action co-representation is tuned to other humans. J. Cogn. Neurosci. 20, 2015–2024 10.1162/jocn.2008.20144 18416679

[B58] Umiltà M. A. Kohler E. Gallese V. Fogassi L. Fadiga L. Keysers C. (2001). I know what you are doing. a neurophysiological study. Neuron 31, 155–165 10.1016/S0896-6273(01)00337-3 11498058

[B59] Van Schie H. T. Mars R. B. Coles M. G. H. Bekkering H. (2004). Modulation of activity in medial frontal and motor cortices during error observation. Nat. Neurosci. 7, 549–554 10.1038/nn1239 15107858

[B60] Van Thiel E. Meulenbroek R. G. Hulstijn W. (1998). Path curvature in workspace and in joint space: evidence for coexisting coordinative rules in aiming. Motor Control 2, 331–351 9758885 10.1123/mcj.2.4.331

[B61] Wada Y. Yamanaka K. Soga Y. Tsuyuki K. Kawato M. (2006). Can a kinetic optimization criterion predict both arm trajectory and final arm posture? Conf. Proc. IEEE Eng. Med. Biol. Soc. 1, 1197–1200 10.1109/IEMBS.2006.260818 17946449

