# Supplementary material for: Motor resonance facilitates movement execution: an ERP and kinematic study
Source: Front Hum Neurosci. 2013 Oct 15;7:646. doi: 10.3389/fnhum.2013.00646 (PMC3796768; doi:10.3389/fnhum.2013.00646)
Supplement: Supplementary Table 1 — Actor's Averaged values for all analyzed parameters for each condition [Mean values ± standard error of the mean (s.e.m.)]. [file DataSheet1.PDF]

Supplementary Table 1: Actor’s Averaged values for all analysed parameters for each condition (Mean values ± standard error of the mean (SEM).

| | | **Total** | **1st part of movement** | | | **2nd part of movement** | | | **Deviation of the extrema of the trajectory (mm)** | | | |
|---|---|---|---|---|---|---|---|---|---|---|---|---|
| **Conditions** | | **Duration t (ms)** | **Vel1 (mm/s)** | **LatPeak 1 (ms)** | **Duration1 (ms)** | **Vel2 (mm/s)** | **LatPeak 2 (ms)** | **Duration2 (ms)** | **Movement deviation** | **Orthogonal deviation** | **Body deviation** | **Reaction Time (ms)** |
| Horizontal | *Congruent* | 2056 ± 91 | 668 ± 53 | 473 ± 25 | 974 ± 50 | 657 ± 44 | 406 ± 17 | 109 ± 43 | 350 ± 10 | 4 ± 3 | 64 ± 7 | 263 ± 23 |
| | *Non-congruent* | 2081 ± 93 | 670 ± 54 | 481 ± 24 | 974 ± 50 | 666 ± 49 | 403 ± 17 | 111 ± 49 | 352 ± 11 | 4 ± 3 | 66 ± 7 | 266 ± 23 |
| Vertical | *Congruent* | 2019 ± 102 | 787 ± 66 | 437 ± 26 | 901 ± 54 | 705 ± 53 | 432 ± 23 | 109 ± 57 | 387 ± 11 | 18 ± 6 | 37 ± 4 | 263 ± 19 |
| | *Non-congruent* | 2030 ± 103 | 794 ± 70 | 430 ± 24 | 893 ± 51 | 707 ± 52 | 438 ± 23 | 111 ± 56 | 389 ± 11 | 21 ± 6 | 38 ± 5 | 261 ± 23 |
| ANOVA (Direction) | | F(1,16)=2,83; ns | F(1,16)=26,85; p<0,0001 | F(1,16)=18,00; p<0,001 | F(1,16)=8,64; p<0,01 | F(1,16)=4,10; ns | F(1,16)=6,36; p<0,02 | F(1,16)=0,03; ns | F(1,16)=42.2; p<0.0001 | F(1,16)=32.1; p<0.0001 | F(1,16)=22.5; p<0.0003 | F(1,16)=0,08; ns |
| ANOVA (Condition) | | F(1,16)=2,84; ns | F(1,16)=0,95; ns | F(2,32)=0,01; ns | F(1,16)=0,29; ns | F(1,16)=1,05; ns | F(2,32)=0,16; ns | F(1,16)=3,72; ns | F(1,16)=0.8; ns | F(1,16)=2.4; ns | F(1,16)=2.1; ns | F(1,16)=0,01; ns |
| ANOVA (Direction*Condition) | | F(1,16)=0,71; ns | F(1,16)=0,01; ns | F(2,32)=1,50; ns | F(1,16)=0,559; ns | F(1,16)=0,28; ns | F(2,32)=1,15; ns | F(1,16)=0,19; ns | F(1,16)=0.5; ns | F(1,16)=0.1; ns | F(1,16)=0.1; ns | F(1,16)=0,22; ns |
